# Syntheses of Sulfo-Glycodendrimers Using Click Chemistry and Their Biological Evaluation

**DOI:** 10.3390/molecules171011877

**Published:** 2012-10-09

**Authors:** Yoshiko Miura, Shunsuke Onogi, Tomohiro Fukuda

**Affiliations:** 1 Department of Chemical Engineering, Graduate School of Engineering, Kyushu University, 744 Motooka, Nishi-ku, Fukuoka 819-0395, Japan; 2 School of Materials Science, Japan Advanced Institute of Science and Technology, 1-1 Asahidai, Nomi, Ishikawa 923-1292, Japan; 3 Department of Applied Chemistry and Chemical Engineering, Toyama National College of Technology, Hongo-campus, 13 Hongo-Machi, Toyama, Toyama 939-8630, Japan

**Keywords:** glycopolymer, dendrimer, click chemistry, amyloidosis

## Abstract

A series of novel glycol-clusters containing sulfonated *N*-acetyl-D-glucosamine (GlcNAc) have been synthesized using click chemistry. Three dendrimers with aromatic dendrons were synthesized using chlorination, azidation and click chemistries. The resulting dendrimers were modified with azide-terminated sulfonated GlcNAc using click chemistry. The sulfonated dendrimers showed affinity for proteins, including the lectin wheat germ agglutinin and amyloid beta peptide (1-42). The dendrimers of **G1** and **G2** in particular showed the largest affinity for the proteins. The addition of the sulfonated GlcNAc dendrimers of **G1** and **G2** exhibited an inhibition effect on the aggregation of the amyloid beta peptide, reduced the β-sheet conformation, and led to a reduction in the level of nanofiber formation.

## 1. Introduction

Saccharides displayed on the surfaces of cells have been the subject of considerable attention because they can play important roles in living systems [1]. Furthermore, they have been related to a variety of different biological activities, including cell-cell adhesion, protein recognition, pathogen infection, and cancer metastasis. For these reasons, materials capable of interfering with the functions of these saccharides are being considered for use in the fabrication of new biomaterials and the development of new drugs [2]. Saccharide-protein interactions, however, are usually too weak to be used as drugs and biomaterials.

Interestingly, it is well known that saccharide-protein interactions can be amplified by multivalency, otherwise known as “the cluster glycoside effect” [3,4]. Clusters of glycosides can amplify the interaction though multiple saccharide binding interactions to the proteins, and increase the binding probability. A variety of different compounds containing multivalent saccharides have been reported to show strong molecular recognition abilities. Furthermore, several saccharide containing substances have been reported such as artificial glycoprotein conjugates, glycopeptides, saccharide-thin layers and saccharide nanoparticles [5,6,7,8,9,10,11]. Of these saccharide containing substances, polymers with saccharide side chains have attracted the most pronounced level of attention because of the large cluster glycoside effects associated with their saccharide-protein interactions. Throughout the remainder of this paper, these polymers will be referred to as “glycopolymers”. These glycopolymers are interesting because they exhibit a strong amplification effect and possess properties making them practical candidates for applications as biomaterials and polymer drug development.

We previously reported the synthesis of a variety of different glycopolymers for lectin recognition, toxin neutralization and cell cultivation [12]. Although these glycopolymers were interesting materials, it was difficult to clarify the detailed mechanism of their interactions, because of the complicated nature of polymer structure resulting from the variable molecular weight and monomer sequence distributions in the copolymers. Of the glycopolymers synthesized and evaluated to date, glycodendrimers possess some of the most interesting characteristics as a consequence of their uniform structure and their extensive molecular recognition ability, with the general expectation that these materials could ultimately be applied as potential polymer drugs [13].

Recently, we reported the synthesis of a sulfonated glycopolymer that interacted with Alzheimer amyloid β (Aβ) and inhibited its aggregation [14,15]. The sulfonated glycopolymer behaved as a mimic of glycosaminoglycans (GAGs) such as heparin and heparan, and interacted with Aβ like GAGs. In the current research, novel glycodendrimers containing a sulfonated saccharide unit were investigated for their ability to inhibit the aggregation of the Aβ (1-42) peptide. Glycodendrimers possess well-defined structures that are generally believed to be more suitable and therefore better suited for use in medicinal applications than liner polymers. The synthesis of the dendrimers in the current report represents one of the key highlights of this research paper because the dendrimers were successfully constructed using click chemistry rather than the tedious multi-step synthetic strategies typically used in the synthesis of these materials [16]. Several dendrimers were synthesized in this way and their inhibitory effects on Aβ aggregation investigated by fluorescence, circular dichroism (CD) spectra, and atomic force microscopy (AFM).

The interactions of these glycodendrimers with wheat germ agglutinin (WGA) were also investigated to clarify the properties of novel cluster glycosides. Since the interactions between the Aβ and GAGs were predominately electrostatic in nature, the synthetic sulfonated glycodendrimer was envisaged to interact with the Aβ in the same way. WGA has a positive net charge in neutral buffer solution [17], and the interaction of the glycodendrimers with WGA was considered to occur though both the molecular recognition of *N*-acetyl-D-glucosamine (GlcNAc) [18] and electrostatic interactions. The interaction with WGA was investigated in view of the Aβ interaction. The ultimate propose of this study was the fabrication of a glycocluster capable of inhibiting Aβ aggregation. 

## 2. Results and Discussion

### 2.1. Synthesis of Dendrimers with 6-Sulfo-GlcNAc

Glycodendrimers of **G1** and **G2** (Figure 1) were synthesized using click chemistry according to the divergent method depicted in Scheme 1, Scheme 2, Scheme 3, Scheme 4, Scheme 5, Scheme 6 and Scheme 7.

**Figure 1:**
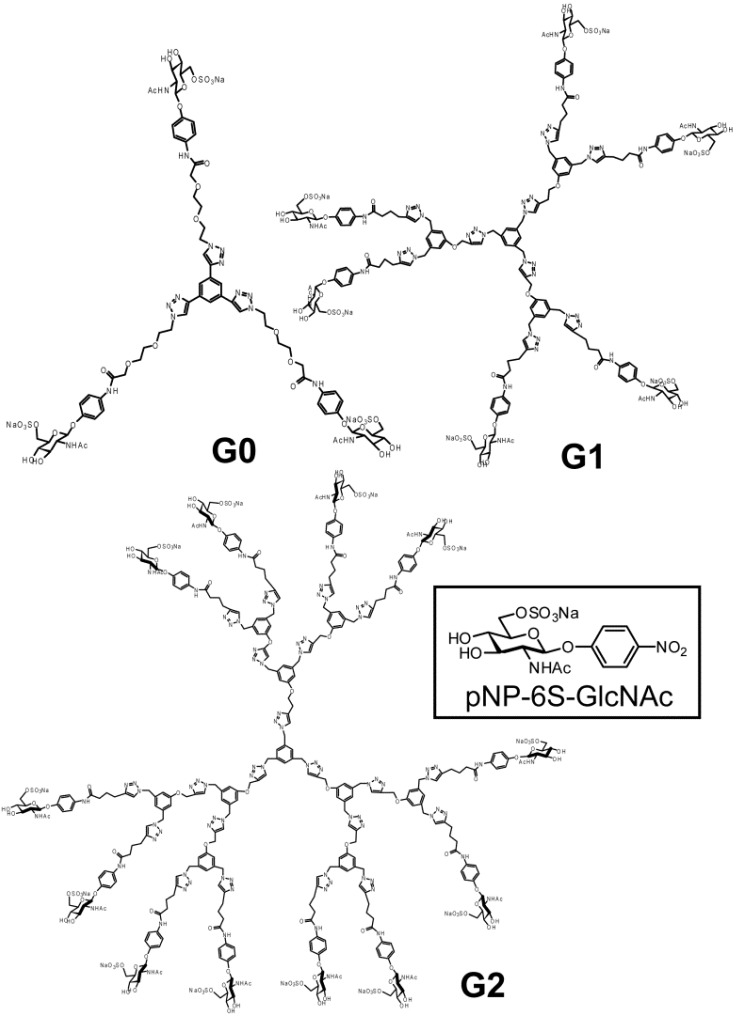
Chemical structures of the saccharide-derivatives.

**Scheme 1:**
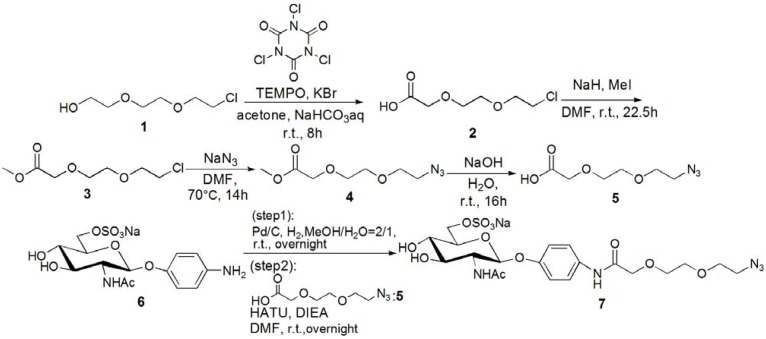
Syntheses of azide-terminated sulfonated GlcNAc **7**.

**Scheme 2:**
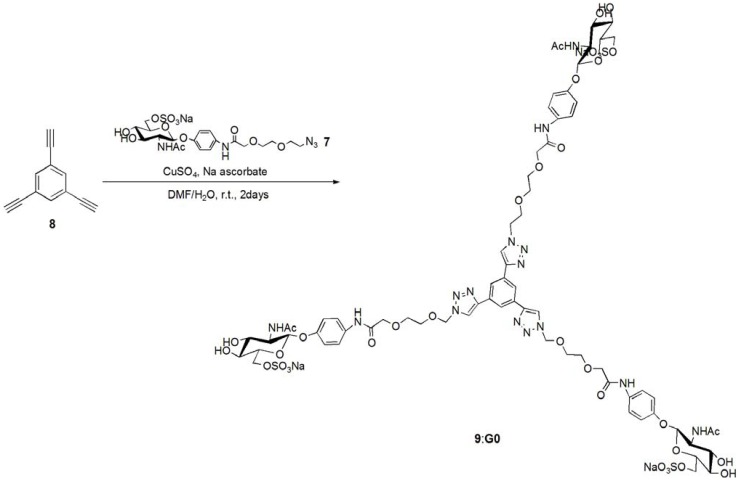
Synthesis of 6-sulfo-GlcNAc trimer **G0**: **9**.

**Scheme 3:**
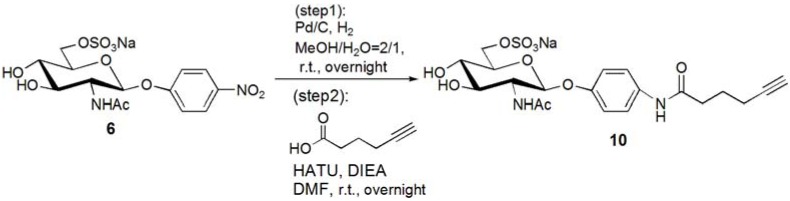
Synthesis of acetylene-terminated sulfonated GlcNAc **10**.

**Scheme 4:**
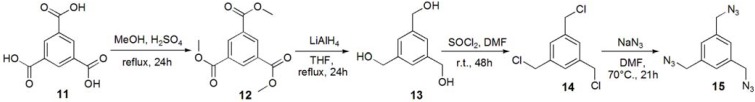
Syntheses of the dendrimer core **15**.

**Scheme 5:**
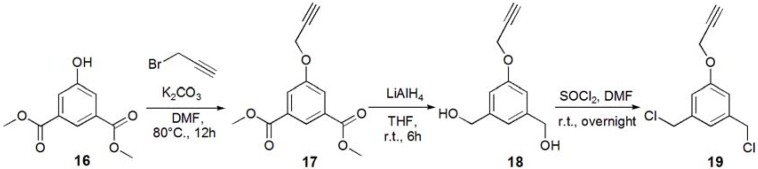
Syntheses of the dendron **19**.

The trimer of 6-sulfo-GlcNAc (**G0**) was synthesized via the connection of azide-terminated 6-sulfo-GlcNAc to 1,3,5-triethynylbenzene, using click chemistry [19,20] (Scheme 1 and Scheme 2). *p*-Aminophenyl-6-sulfo-D-GlcNAc was synthesized according to a method previously reported in the literature [14,15] and subsequently connected to *N*-phenylhex-5-ynamide using 2-(7-aza-1*H*-benzotriazole-1-yl)-1,1,3,3-tetramethyl uranium hexafluorophosphate (HATU) and *N*,*N*-diisopropylethylamine (DIEA) (Scheme 3). Starting from 5-hydroxyisophthalic acid, the dendron of 1,3-bis(chloromethyl)-5-(prop-2-yn-1-yloxy)benzene was synthesized by sequential alkylation, hydrogenation and chlorination reactions, with the resulting dendron being attained in high yield (**G1-N_3_**: 93%, **G2-N_3_**: 56%) (Scheme 4 and Scheme 5). 

**Scheme 6:**
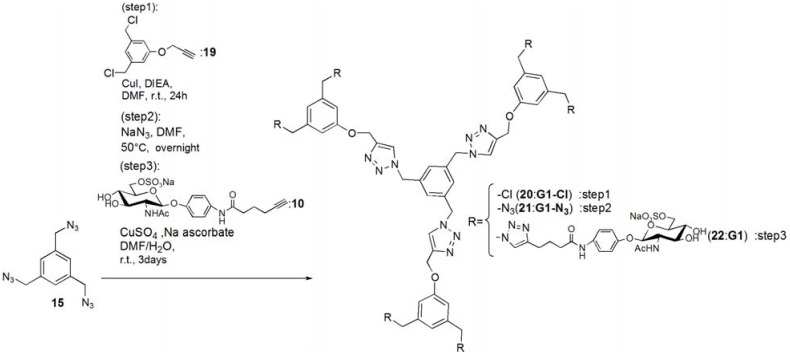
Syntheses of 6-sulfo-GlcNAc hexamer **G1**: **22**.

**Scheme 7:**
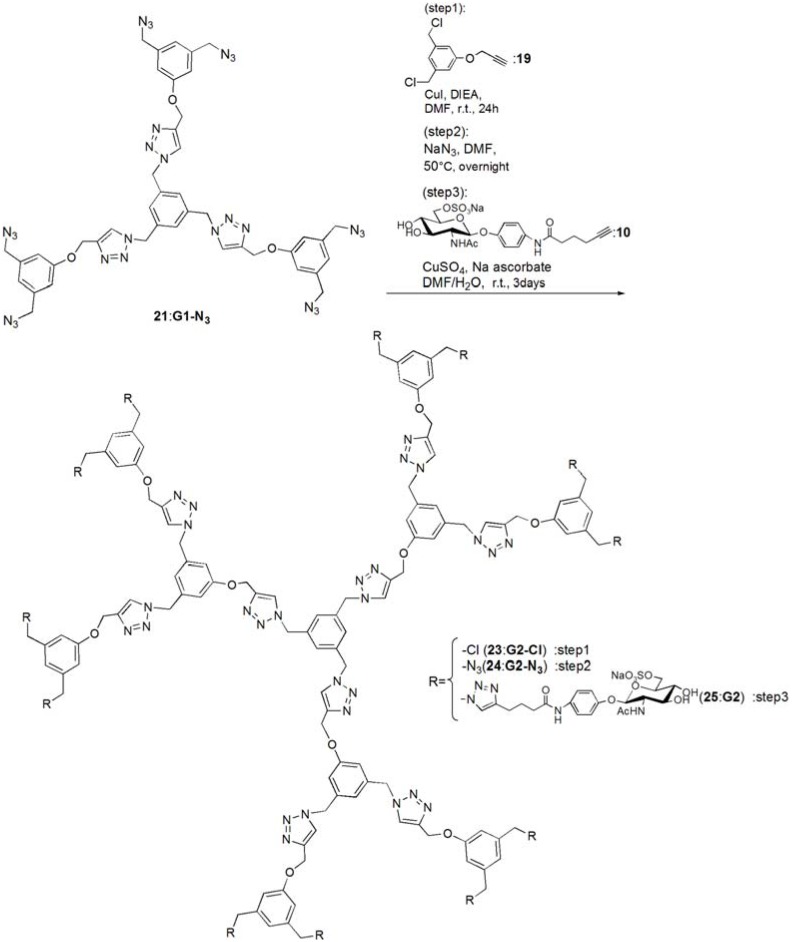
Syntheses of 6-sulfo-GlcNAc dodecamer **G2**: **25**.

Glycodendrimers of **G1** and **G2** were obtained by click chemistry in a modest yield (**G1**: 28%, **G2**: 21%) (Scheme 6 and Scheme 7). 1,3,5-Tris(azidomethyl)benzene was used as the core of the dendrimer and was itself derived from 1,3,5-trimesic acid via sequential hydrogenation, chlorination and azidation reactions (Scheme 3). The structures of the compounds were confirmed by ^1^H and ^13^C-NMR, electrospray ionization mass spectroscopy (ESI-MS) and matrix-assisted laser desorption ionization and time of flight mass spectroscopy (MALDI-TOF-MS). Detailed syntheses and characterizations are provided in the Experimental Section.

### 2.2. Lectin Recognition Ability

**Figure 2:**
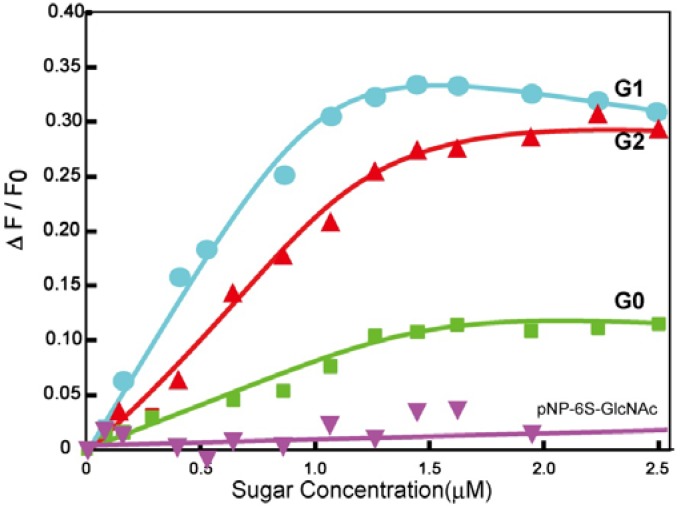
Fluorescence intensity changes of the FITC-WGA with varying sugar concentrations of the sulfonated GlcNAc derivatives.

Although the interaction mechanism of WGA is different from that of Aβ, the synthesized glycodendrimers were evaluated in a lectin recognition assay to estimate their biological ability (Figure 2) [19]. 

Fluorescence quenching of the fluorescein isothiocyanate (FITC)-WGA complex occurred upon the addition of the glycodendrimers [21]. The quenching behavior was analyzed using a Scatchard plot. The binding constants (K_a_) of **G0**, **G1** and **G2** were found to be 6.56 × 10^5^, 5.55 × 10^6^ and 2.12 × 10^6^ (M^−1^), respectively, with the affinity constants therefore being of the order **G1** > **G2** >> **G0**. In contrast to WGA, the monomeric sulfonated GlcNAc did not show the same detectable affinity for lectin, and the addition of the glycodendrimers to FITC-bovine serum albumin did not induce fluorescence quenching. The results showed that the glycodendrimer of **G1** and **G2** had the ability to amplify the protein-saccharide interactions, indicating that the protein-saccharide interactions were specific to a certain extent. **G0** did not show a high level of affinity for WGA, suggesting the potential for weak interactions to proteins including Aβ.

**Figure 3:**
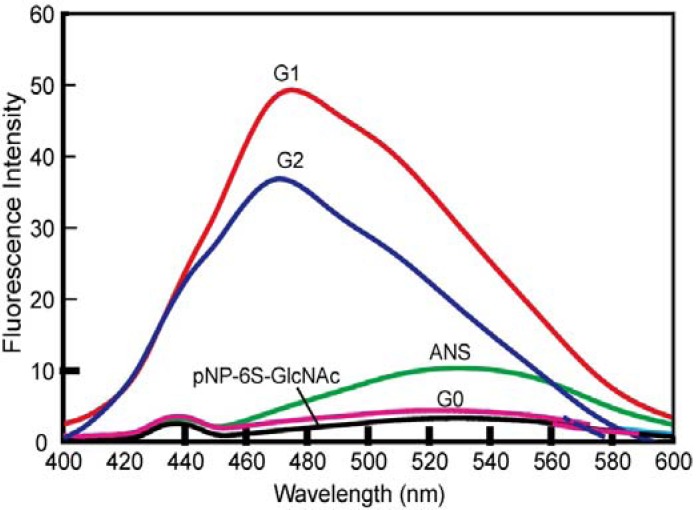
Fluorescence spectra of ANS (20 μM) with sugar derivatives (66.7 μM) in PBS buffer.

The hydrophobic domains of the dendrimes of **G1** and **G2** were analyzed by fluorescence of 8-anilino-1-naphthalene sulfonate (ANS) (Figure 3). The solution of **G1** showed a larger blue shift in its emission spectra than that of **G2**, whereas the solution of **G0** did not show any blue shift. 

The hydrophobicity of dendrimer suggested that they would possess self-assembling properties in solution. Interestingly, the larger fluorescence of **G1** *versus* that of **G2** indicated the larger self-assembling properties of **G1** in the aqueous solution. In addition, the glycodendrimers induced the formation of WGA aggregates. The diameters of WGA-glycodendrimer aggregates in the presence of WGA (1.00 μM) were 752, 496 and 384 nm, respectively, with **G1**, **G2**, and **G0** (data not shown). 

Multivalent GlcNAc generally showed a strong interaction based on the cluster glycoside effect [22], which was similar to the interaction observed for **G1** and **G2**. The positive net charge of WGA also contributed to its interactions with **G1** and **G2** in an electrostatic manner. The sizes of the dendrimers by dynamic light scattering (DLS) became semi-micro in order in the presence of the WGA because of the cross-linking between the WGA and the dendrimer that occurred as a consequence of molecular recognition. The affinity of **G1** was larger than that of **G2**, although the structure of **G2** apparently exhibited a greater degree of multivalency. In addition, considering that the distance between the sugar binding sites was 1.5–5 nm in WGA [23,24], the sugar distance of **G2** was better suited to accommodate WGA than that of **G1**. The affinities of dendrimers, however, showed an opposing trend. Taking the ANS results into account, **G1** formed larger glycoside clusters than **G2** through self-assembly, which resulted in the larger affinity for WGA. Given that the self-assembly plays an important role in the cluster glycoside effect of dendrimers, the affinity between WGA and dendrimer was considered to be dynamic including the cross linking and statistical re-binding aspects of the process. The results suggested that the glycodendrimers of **G1** and **G2** provided glycoside clusters suitable for binding WGA and Aβ.

### 2.3. Inhibitory Effect of Glycodendrimer on Aβ (1-42) Aggregation

**Figure 4:**
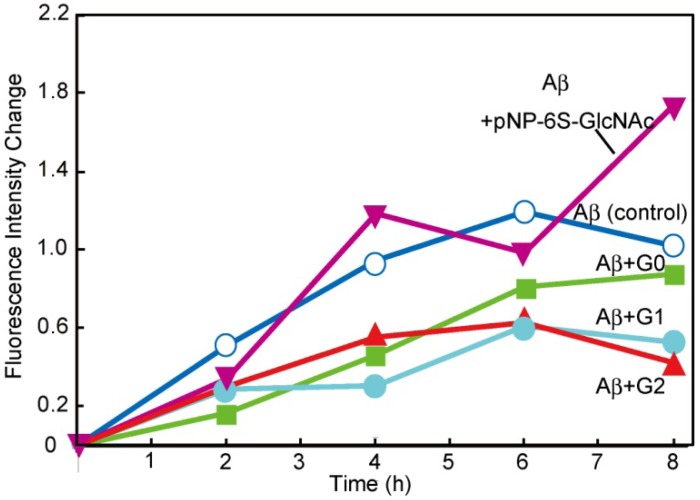
Time course of the fluorescence change in ThT with Aβ (1-42) (20 μM) and sugar derivatives (200 μM) in the phosphate buffer.

The interactions of the glycodendrimers with Aβ were investigated. The aggregation properties of Aβ (1-42) were analyzed by fluorescence spectroscopy with thioflavin T (ThT) [14,15]. It is known that ThT has an affinity for amyloid fibril and that the fluorescence of ThT increases when ThT is attached to amyloid fibrils. In the current experiment, the degree of amyloid formation was monitored by the fluorescence of ThT [25]. The level of amyloidosis was examined over an 8 h period and the fluorescence intensity following this period was evaluated as 1.0. The aggregation behaviors with various sugar additives are shown in Figure 4. 

The monomeric 6-sulfonated GlcNAc did not exhibit an inhibitory effect on Aβ aggregation, as we previously reported [14,15]. In contrast, the addition of sulfonated glycodendrimer modestly inhibited the aggregation of Aβ. The degree of inhibition was in the order **G1** = **G2** > **G0** >> monomer. The addition of dendrimer inhibited the aggregation by approximately 50% relative to the level of inhibition observed in the absence of the additives.

The dendrimers of **G1** and **G2** were effective inhibitors of Aβ aggregation, whereas the inhibitory effect of **G0** was insufficient. The observed order of the inhibitory effect was similar to that observed for the lectin recognition. The results suggested that **G1** and **G2** displayed saccharides in a favorable manner for effective protein affinity. The molecular recognition of the saccharides by lectin was affected by the cluster glycoside effect, which was controlled by the spatial arrangement of the saccharides in the dendrimers or the self-assembled dendrimers. The appropriate glycoside clusters for WGA could also become a better inhibitor for Alzheimer amyloid aggregation. 

It has been reported that GAGs bind to Aβ via electrostatic interactions with the basic segment 13HHQK16 [26]. Glycopolymers containing sulfonated saccharides were considered to interact with Aβ in the same way via electrostatic interactions [14,15]. Glycodendrimers are also considered to bind to Aβ via the same electrostatic interactions. Although the glycodendrimer **G1** showed the largest binding properties, its binding to Aβ was almost identical to that of **G2**. Although the structures of WGA and Aβ are totally different, it was considered that the larger cluster of the glycosides would show high levels of affinity that were similar to those observed in their interaction with WGA.

Multivalent sulfonated groups have been reported to interact with Aβ with GAGs [26], 6-sulfoGlcNAc polymer [14,15], sulfonated Glc polymer [27], and the polymer with sulfonic acid [28]. It is obvious that the sulfonic acid interact with Aβ via electrostatic interaction. The structural specificity and the mechanism of Aβ interactions are under investigation in our group.

### 2.4. Conformation Analysis of Aβ with CD Spectra

**Figure 5:**
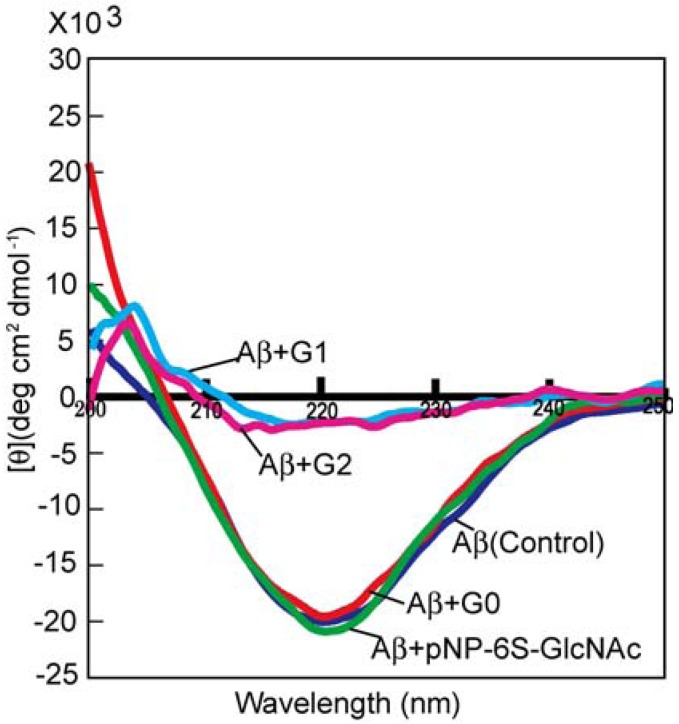
CD spectra for Aβ (1-42) (20 μM) in the presence of the sugar dendrimer additives (200 μM) in the phosphate buffer.

The conformation of Aβ was investigated in the presence of the sulfonated glycodendrimer (Figure 5). In the absence of the sugar additives, Aβ showed the negative cotton effect around 220 nm, suggesting β-sheet conformation [29]. Aβ also showed β-sheet structure in the presence of the monomeric sulfonated saccharide and G1. In contrast, the additions of G1 and G2 led to significant changes in the conformation, with the negative cotton effect observed around 220 nm almost disappearing entirely. The addition of G0 did not lead to a decrease in the negative cotton effect, suggesting that the interaction with Aβ was weak. 

CD spectra recorded in the presence of the sulfonated glycodendrimer indicated that its addition led to the inhibition of the aggregation and conformation change of Aβ. Since the amyloidosis relates to changes in the conformation and aggregation, these results were in good agreement with the Th-T experiments. The inhibition of Aβ aggregation was also evaluated with the sulfonated glycopolymer. The results revealed that the sulfonated glycoside clusters inhibited oligomer formation during the early stages of the amyloidosis. It was also considered that the sulfonated glycodendrimer interacted with Aβ and inhibited the formation of the Aβ oligomer [14].

### 2.5. Morphology Observation of Aβ

**Figure 6:**
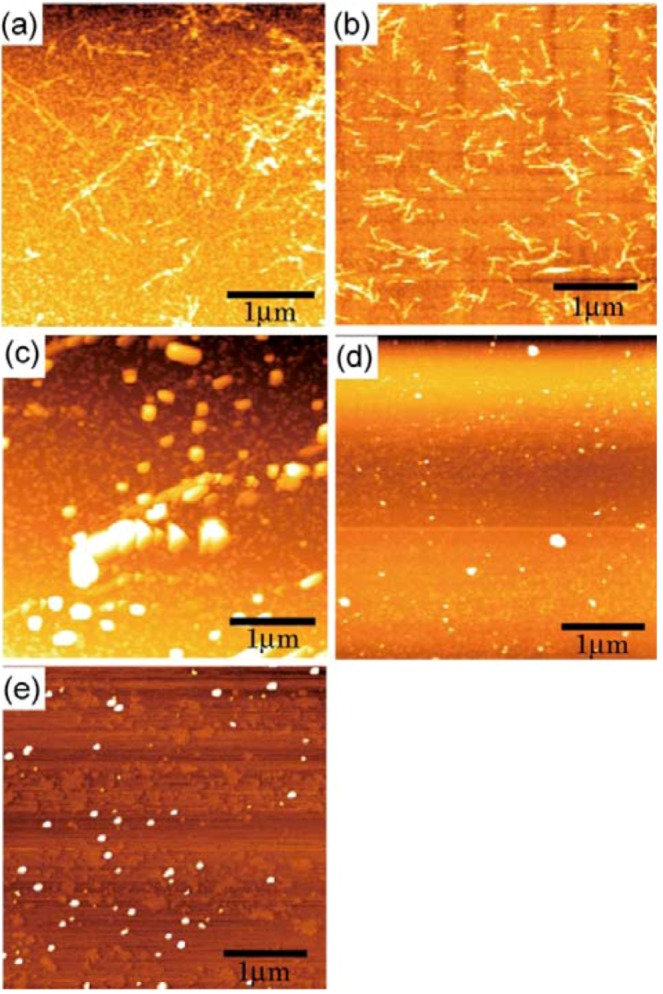
AFM observations of Aβ (1-42) (20 μM) (**a**) in the absence of an additive, (**b**) in the presence of pNP-6S-GlcNAc(200 μM), (**c**) in the presence of G0 (200 μM), (**d**) in the presence of G1 (200 μM), and (**e**) in the presence of G2 (200 μM).

To develop an understanding of the amyloidosis and the properties of the inhibitors, it was necessary to develop an understanding of the Aβ morphology, because the results from the ThT fluorescence and CD spectra only provided an indirect reflection of the protein amyloidosis. The properties of the amyloid, including its cytotoxicity, were strongly related to the size and shape of the aggregates, and so the AFM measurements were indispensable in evaluating these properties [30]. Aβ spontaneously formed nanofiber upon incubation, with widths of 45-70 nm and length of only a few micrometers (Figure 6a). Aβ also formed nanofibers in the presence of the monomeric saccharide with widths of 35–70 nm, although the fibers were shorter in length at less than 1 μm (Figure 6b). In contrast, the additions of **G1** and **G2** led to significant changes in the morphology of Aβ. Nanofibers of the Aβ amyloid were not observed at all in the presence of **G1** and **G2** (Figure 6d,e). Interestingly, the addition of the sulfonated trimer of **G0** induced the largest aggregates (Figure 6c).

The morphology of Aβ correlated well with the results of the ThT and CD analyses. Aβ formed nanofibers in the absence of any additives. AFM analysis revealed that the monomeric 6-sulfonated GlcNAc showed the lowest level of Aβ aggregation inhibition, and this result was consistent with the results of the ThT fluorescence and CD spectra. In contrast, the AFM results indicated that the dendrimers of **G1** and **G2** showed the highest levels of Aβ aggregation inhibition, which were in good agreement with the results of the ThT and CD spectra. The formation of large aggregates was inhibited by the strong interaction with the glycodendrimer. 

**Figure 7:**
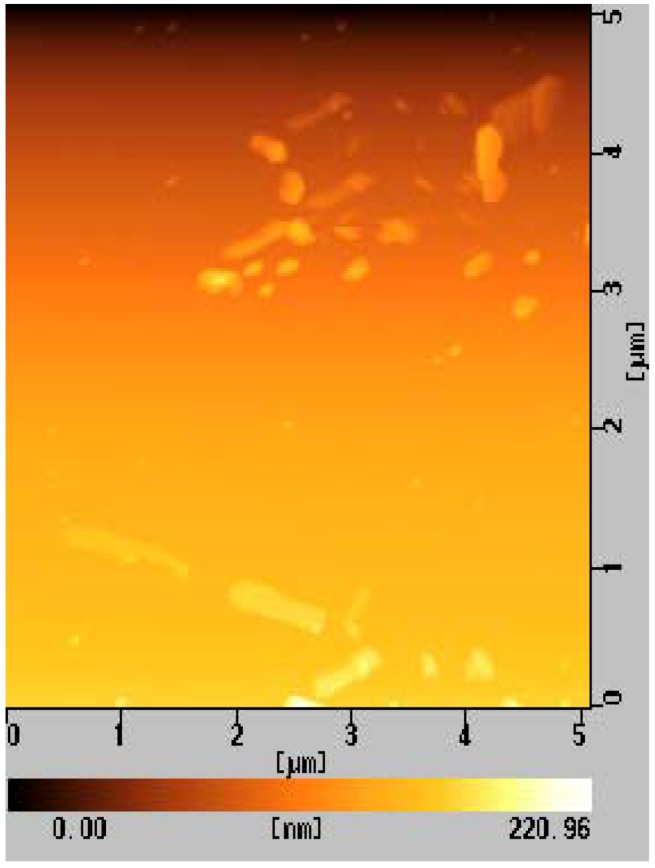
AFM observation of G0 (200 μM).

For **G0** (trimer of 6-sulfonated GlcNAc), the AFM results indicated that the material only exerted a minor impact on the inhibition of Aβ aggregation, with the result being consistent with the ThT and CD results. Furthermore, the aggregates in this case were much larger than those formed in the absence of the additives. The shape of the aggregates was different from the nanofiber of the amyloid. The morphology of **G0** was measured in the absence of Aβ and the results indicated that **G0** formed small aggregates (Figure 7) even though the hydrophobicity by ANS fluorescence was small. 

The morphology of Figure 6c was considered to be representative of a mixture of Aβ and **G0** aggregates. Since the interaction of Aβ with **G0** was weaker than its interactions with **G1** and **G2**, the nanofiber formation of Aβ aggregates was not hindered. **G0** was envisaged to have behaved as a glue, effectively bringing Aβ units together to yield the observed large objects. The morphology in this particular experiment was determined to be mixture of Aβ, Aβ-dendrimer and dendrimer aggregates. In spite of this, it was expected that the cytotoxicity of Aβ would be reduced by the addition of **G0** because of the resulting reduction in the number of toxic Aβ aggregates.

## 3. Experimental

### 3.1. Materials

The following reagents were used as received: *N*-acetylglucosamine, copper(0) powder, copper(II) sulfate, copper(I)iodide, *N*,*N*-dimethylformamide (DMF), chloroform (CHCl_3_), ethyl acetate (EtOAc), *n*-hexane, magnesium sulfate (MgSO_4_), methanol (MeOH), methylene chloride (CH_2_Cl_2_), potassium bromide (KBr), potassium carbonate (K_2_CO_3_), 2-propanol, sodium L-ascorbate, sodium azide (NaN_3_), sodium bicarbonate (NaHCO_3_), sodium carbonate (Na_2_CO_3_) sodium hydride (NaH), sodium hydroxide (NaOH), tetrahydrofuran (THF), thioflavin-T(ThT), thionyl chloride (SOCl_2_), (Kanto Chemical Co., Inc., Tokyo, Japan), 2-[2-(2-chloroethoxy)ethoxy]ethanol, DIEA, 5-hexynoic acid, propargyl bromide, 2,2,6,6-tetramethylpiperidine 1-oxyl (TEMPO), trichloroisocyanuric acid, 1,3,5-triethynylbenzene, trimesic acid (TCI Co., Tokyo, Japan), amyloid β protein (human, 1-42) (Aβ (1-42)), HATU (Peptide Institute Inc., Osaka, Japan), palladium/charcoal (Pd/C) (Merck & Co., Inc., Whitehouse Station, NJ, USA), and deuterated solvents (CDCl_3_, CD_3_OD, DMF-*d_7_* and D_2_O), dimethyl 5-hydroxyisophtalate, and lithium alminium hydride (LiAlH_4_) (Sigma-Aldrich, St. Louis, MO, USA). 6-sodium sulfo-*N*-acetyl-β-D-glucosamine (**6**) was synthesized according to the previous literature [5,14].

### 3.2. Measurements

The identities of the compounds were confirmed by the following methods. Both ^1^H-NMR (300 MHz) and ^13^C-NMR (75 MHz) spectra were recorded using a Varian Gemini 2000 spectrometer and ^1^H-NMR (500 MHz) spectra was recorded using a Varian UNITY 500plus spectrometer, equipped with a Sun workstation, respectively. The spectra were measured in CDCl_3_, CD_3_OD, DMF-d_7_ and D_2_O solvents at room temperature. Mass spectra were measured on a MALDI-TOF-MS (Voyager, Applied Biosystems, Foster City, CA, USA) and ESI-MS (LCQ Deca xp, Thermo Fisher Scientific, Waltham, MA, USA). CD spectra were recorded using a JASCO J-720 spectrometer (JASCO, Tokyo, Japan). AFM measurement was carried out using SPA400 instrument (Seiko Instruments Inc., Tokyo, Japan) with a 40 N cantilever. Fluorescence spectra were measured on a JASCO FP-6500 (JASCO) at 25 °C. DLS for the GNPs was determined using a Zetasizer 3000 (Sysmex, Kobe, Japan).

### 3.3. Syntheses

*[2-(2-Chloroethoxy)ethoxy]acetic acid *(**2**). [2-(2-Chloroethoxy)ethoxy]ethanol (**1**, 2.20 g, 13.0 mmol) was dissolved in acetone (30 mL), and 15% NaHCO_3_ aq. was added to the solution at 0 °C. KBr (0.312 g, 2.60 mmol) and TEMPO (0.040 g, 0.040 mmol) were added to the solution, and trichloroisocyanuric acid (6.10 g, 26.2 mmol) was added dropwise. The solution was allowed to warm up to room temperature, and stirred for 8 h. The reaction was confirmed by TLC (EtOAc–*n*-hexane = 3:1), and 2-propanol (10 mL) was added to quench the reaction. The reaction mixture was filtrated with Celite, and neutralized with sat. Na_2_CO_3_ aq. The solution was acidified with 1 N HCl, and extracted with CHCl_3_. The organic phase was dried over MgSO_4_, and MgSO_4_ was removed by filtration. The solution was evaporated to yield yellow oil; Yield 1.94 g, 88.6%. ^1^H-NMR (300 MHz, r.t., CDCl_3_): δ/ppm 3.58–3.60 (m, 2H, CH_2_), 3.64–3.68 (m, 2H, CH_2_), 3.69–3.70 (m, 2H, CH_2_), 3.71–3.76 (m, 2H, CH_2_), 4.18 (s, 2H, CH_2_). IR wavenumber [cm^−1^]: 3470 (OH) 1734 (C=O) 1112 (C-O). ESI-MS (negative): 181.2 [M−H]^−^.

*Methyl 2-[2-(2-chloroethoxy)ethoxy]acetate *(**3**). Compound **2** (0.312 g, 1.78 mmol) was dissolved in DMF (2 mL), and NaH (0.530 g, 2.22 mmol) was added to the solution at 0 °C. After stirring for 30 min, MeI (0.649 g, 3.33 mmol) was added to the solution. The reaction mixture was stirred for 22.5 h at room temperature. The reaction was confirmed by TLC (EtOAc–*n*-hexane = 1:3). The solution was evaporated, and the residue was dissolved in CHCl_3_. The solution was washed with 1 N HCl, sat. NaHCO_3_ aq. and brine. The solution was dried over MgSO_4_, and MgSO_4_ was removed by filtration. The residue was purified by column chromatography (EtOAc–*n*-hexane = 1:3) to yield colorless oil; Yield 0.242 g, 74.4%. ^1^H-NMR (300 MHz, r.t., CDCl_3_): δ/ppm 3.62 (t, 2H, *J* = 6 Hz, *J* = 6 Hz, CH_2_), 3.71–3.72 (m, 2H, CH_2_), 3.72–3.74 (m, 2H, CH_2_), 3.73 (s, 3H, CH_3_), 3.74–3.76 (m, 2H, CH_2_), 4.16 (s, 2H, CH_2_). ESI-MS (positive): 219.1 [M+Na]^+^.

*Methyl 2-[2-(2-azidoethoxy)ethoxy]acetate *(**4**). Compound **3** (0.242 g, 1.31 mmol) was dissolved in DMF (15 mL). NaN_3_ (0.511 g, 7.86 mmol) was added to the solution, and stirred for 14 h at 70 °C. The reaction was confirmed by TLC (EtOAc–*n*-hexane = 1:3). NaN_3_ was removed by filtration, and the solution was evaporated. The residue was dissolved in CHCl_3_, and the solution was washed with 1 N HCl, sat. NaHCO_3_ aq. and brine. The organic phase was dried over MgSO_4_, and MgSO_4_ was removed by filtration. The solution was evaporated to yield colorless oil; Yield 0.220 g, 87.8%. ^1^H-NMR (300 MHz, r.t., CDCl_3_): δ/ppm 3.38 (t, 2H, *J* = 5 Hz, *J* = 5 Hz, CH_2_), 3.65–3.67 (m, 2H, CH_2_), 3.67–3.67 (m, 2H, CH_2_), 3.70–3.72 (m, 2H, CH_2_), 3.73 (s, 3H, CH_3_), 4.16 (s, 2H, CH_2_). ESI-MS (positive) 226.1 [M+Na]^+^.

*[2-(2-Azidoethoxy)ethoxy]acetic acid *(**5**). Compound **4** (0.220 g, 1.15 mmol) was dissolved in 1 N NaOHaq (15 mL), and the solution was stirred for 16 h. The reaction was confirmed by TLC (EtOAc–*n*-hexane = 1:3). The solution was acidified with 1 N HCl, and extracted with CHCl_3_. The solution was dried over MgSO_4_, and MgSO_4_ was removed by filtration. The solution was evaporated to yield colorless oil; Yield 0.172g, 84.4%. ^1^H-NMR (300 MHz, r.t., CDCl_3_): δ/ppm 3.41 (t, 2H, *J* = 4.8 Hz, *J* = 5.1 Hz, CH_2_), 3.67–3.69 (m, 2H, CH_2_), 3.70–3.71 (m, 2H, CH_2_), 3.75–3.78 (m, 2H, CH_2_), 4.16 (s, 2H, CH_2_). ESI-MS (negative): 188.6 [M−H]^−^.

*p-N-[2-(2-Azidoethoxy)ethoxy]amidophenyl-2-acetamido-2-deoxy-6-sulfo-β-D*-*glucopyranoside *(**7**)*.* Compound **6** was synthesized by following the previous method [5,14]. Next **6** (0.142 g, 0.320 mmol) was dissolved in MeOH–H_2_O = 2:1 (10 mL). Pd/C (20 mg) was added to the solution, and the solution was stirred overnight under H_2_. Pd/C was removed by filtration, and the solution was evaporated. The residue was dissolved in DMF (5 mL), and **5** (86.2 mg, 0.456 mmol) was added to the solution. HATU (185 mg, 0.487 mmol) and DIEA (83 μL, 0.487 mmol) were added to the solution, and the solution was stirred overnight. The solution was evaporated, and the residue was purified by column chromatography (H_2_O–MeOH = 5:1) to yield a brown solid; Yield 0.160 g, 83.2%. ^1^H-NMR (500 MHz, r.t., D_2_O): δ/ppm 2.09 (s, 3H, OCOCH_3_), 3.36–3.39 (m, 2H, CH_2_), 3.48 (t, 1H, *J*_H3-H4_ = 9.0 Hz, *J*_H4-H5_ = 9.5 Hz, H4), 3.54 (t, 1H, *J*_H2-H3_ = 10.0 Hz, *J*_H3-H4 _= 9.5 Hz, H3), 3.57–3.62 (m, 2H, CH_2_), 3.66–3.68 (m, 2H, CH_2_), 3.71–3.73 (m, 2H, CH_2_), 3.74–3.75 (m, 1H, H5), 3.88 (t, 1H, *J*_H1-H2_ = 9.5 Hz, *J*_H2-H3_ = 9.5 Hz, H2), 4.11 (s, 2H, CH_2_), 4.13 (dd, 1H, *J*_H5-H6proR_ = 4.0 Hz, *J*_H6proR-H6proS_ = 6.0 Hz, H6proR), 4.28 (d, 1H, *J *= 10 Hz H6proS), 5.01 (d, *J *= 8.5 Hz, H1), 6.98(d, 2H, *J*_o-m_=7.0 Hz, Ph-o), 7.27 (d, 2H, *J*_o-m_ = 9.0 Hz, Ph-m). ESI-MS (negative): 421.1 [M−Na]^−^. 

*pNP 6-Sulfo-GlcNAc trimer *(**G0**:**9**). Compound **7** (91.8 mg, 0.157 mmol) and 1,3,5-triethylbenzene (**8**) (5.12 mg, 0.0341 mmol) were dissolved in DMF (20 mL). To the solution, sodium ascorbate (12.2 mg, 0.6 eq.) and CuSO_4_ (4.91 mg, 0.3 eq.) was added. The solution was stirred for 2 days, and the reaction was confirmed by reversed phase TLC (H_2_O–MeOH = 5:1). Copper was removed by centrifugation, and the residue was purified by column chromatography (H_2_O–MeOH = 5:1) to yield colorless solid; Yield 0.0320g, 49.2%. ^1^H-NMR (300 MHz, r.t., D_2_O):δ/ppm 1.86(s, 9H, CH_3_), 3.41–3.47 (overlap, 6H, CH_2_), 3.41–3.47 (overlap, 3H, H4), 3.52–3.57 (overlap, 3H, H3), 3.52–3.57 (overlap, 12H, CH_2_ × 2), 3.66 (broad, 3H, 5H), 3.66 (broad, 6H, CH_2_), 3.75–3.77 (overlap, 3H, H2), 3.77 (s, 6H, CH_2_), 3.98 (s, 6H, NH), 4.05–4.07 (m, 3H, H6proR), 4.16 (d, 3H, *J *= 11.5 Hz H6proS), 4.61–4.62 (overlap, 3H, H1), 6.47 (d, 6H, *J*_o-m_ = 9.0 Hz, Ph-o), 6.77 (d, 6H, *J*_o-m_ = 8.5 Hz, Ph-m), 7.53 (s, 3H, Ar), 8.18 (s, 3H, C=CH). ^13^C-NMR (75 MHz, r.t., D_2_O): δ/ppm 21.6, 49.5, 53.7, 54.8, 66.2, 67.8, 68.7, 68.8, 69.2, 72.9, 73.3, 99.3, 116.1, 121.2, 121.7, 122.6, 130.0, 130.6, 153.2, 169.2, 174.0. ESI-MS: *m/z* 976.1 [M+2Na]^2+^, (LCMS, negative) *m/z* 822.9 [M−3Na]^3−^.

*p-N-(5-Hexynoic)amidophenyl-2-acetoamido-2-deoxy-6-sulfo-β-D-glucopyranoside *(**10**). Compound **6** was dissolved in MeOH–H_2_O = 2:1. Pd/C was added to the solution, and the solution was stirred overnight under H_2_. Pd/C was removed by filtration, and the solution was evaporated. The residue, *p*-aminophenyl-2-acetoamido-2-deoxy-6-sulfo-β-D-glucopyranoside (0.353 g, 0.853 mmol) was dissolved in DMF (20 mL), and 5-hexynoic acid (0.143 g, 1.28 mmol) was added to the solution at 0 °C. HATU (0.486 g, 1.28 mmol) and DIEA (217 μL, 1.28 mmol) was added to the solution, and the solution was stirred overnight. DMF was evaporated, and the residue was purified by reversed phase column chromatography (H_2_O–MeOH = from 5:1 to 2:1) to yield white solid; Yield 0.366g, 81.6%. ^1^H-NMR (500 MHz, r.t., D_2_O): δ/ppm 1.74 (t, *J *= 7.0Hz, 2H, CH_2_), 1.90(s, 3H, OCOCH_3_), 2.15–2.18 (m, 2H, CH_2_), 2.25 (t, *J *= 2.5Hz, 1H, C≡CH), 2.36–2.39 (m, 2H, CH_2_), 3.47 (t, 1H, *J*_H3-H4_ = 9.5 Hz, *J*_H4-H5_ = 9.5 Hz, H4), 3.52–3.55 (m, 1H, H3), 3.70–3.73 (m, 1H, H5), 3.87 (t, 1H, *J*_H1-H2_ = 8.5Hz, *J*_H2-H3_ = 10 Hz, H2), 4.11 (dd, 1H, *J*_H5-H6proR_ = 5.5Hz, *J*_H6proR-H6proS_ = 5.0 Hz, H6proR), 4.25 (d, 1H, *J *= 11.5 Hz, H6proS), 4.99 (d, *J *= 8.5 Hz, H1), 6.93 (d, 2H, *J*_o-m_ = 9.5 Hz, Ph-o), 7.27 (d, 2H, *J*_o-m_ = 9.5 Hz, Ph-m).

*Trimethyl 1,3,5-benzenetricarboxylate *(**12**). Trimesic acid (**11**, 8.00 g, 38.1 mmol) was dissolved in MeOH (140 mL), and conc. H_2_SO_4_ (2 mL) was added to the reaction mixture. The solution was refluxed for 24 h, and the solution was evaporated. The residue was dissolved in CHCl_3_, and washed with sat. Na_2_CO_3_aq. The solution was dried with MgSO_4_, and removed by filtration. The solution was evaporated to obtain white solid; Yield 9.09 g, 94.7%. ^1^H-NMR (300 MHz, r.t., CDCl_3_): δ/ppm 3.96 (s, 9H, OMe), 8.83 (s, 3H, Ar).

*1,3,5-Trihydroxymethylbenzene *(**13**). Compound **12** (1.00 g, 3.97 mmol) was dissolved in THF (70 mL). LiAlH_4_ (35.7 mmol) was added to the solution, and the solution was refluxed for 24 h. The reaction was confirmed by TLC (EtOAc–*n*-hexane=1:1), and water was added to the reaction in order to quench the reaction. The solution was dried over MgSO_4_ and evaporated to yield white solid; Yield 0.571 g, 85.6%. ^1^H-NMR (300 MHz, r.t., CDCl_3_): δ/ppm 4.60 (s, 6H, CH_2_), 7.25 (s, 3H, Ar).

*1,3,5-Tribenzylchloride *(**14**)*.* Compound **13 **(0.200 g, 1.19 mmol) was dissolved with SOCl_2_ (5 mL) at 0 °C and to the solution the catalytic amount of DMF was added. The solution was stirred for 48 h at room temperature. The reaction was confirmed by TLC (EtOAc–*n*-hexane = 1:1). The reaction mixture was poured into ice-water, and extracted with CHCl_3_. The organic phase was washed with water, and dried over MgSO_4_. The solution was evaporated to yield white solid; Yield 0.241 g, 93.9%. ^1^H-NMR (300 MHz, r.t., CDCl_3_): δ/ppm 4.57 (s, 6H, CH_2_), 7.36 (s, 3H, Ar).

*1,3,5-Tribenzylazide *(**15**). Compound **14** (0.214 g, 0.964 mmol) was dissolved in DMF (10 mL). NaN_3_ (1.13 g, 17.4 mmol, 18 eq.) was added to the solution, and the solution was stirred at 70 °C for 21 h. The solution was evaporated, and the residue was dissolved with chloroform. The solution was washed with 1 N HCl aq., sat. NaHCO_3_, and brine. The solution was dried over MgSO_4_. The solution was evaporated to yield colorless oil; Yield 0.218 g, 93.0%. ^1^H-NMR (300 MHz, r.t., CDCl_3_): δ/ppm 4.38 (s, 6H, CH_2_), 7.23 (s, 3H, Ar).

*1-(3-Acetyl-5-prop-2-ynyloxy-phenyl)ethanone *(**17**). Dimethyl 5-hydroxyisophtalate (**16**, 5.01 g, 23.8 mmol) and K_2_CO_3_ (93 g, 35.7 mmol, 1.5 eq.) were dissolved in DMF (100 mL), and the solution was degassed with bubbling of N_2_. The solution was heated up to 80 °C, and propargyl bromide (2.80 mL, 35.7 mmol, 1.5 eq.) was added under N_2._ The solution was stirred for overnight. The solution was allowed to be cooled to room temperature. K_2_CO_3_ was removed by filtration, and the solution was evaporated. The residue was recrystallized in EtOH to yield white solid; Yield: 4.52 g, 76.5%. ^1^H-NMR (300 MHz, r.t., CDCl_3_): δ/ppm 2.53 (t, *J *= 2.1 Hz, *J *= 2.4 Hz, 1H, C≡CH), 3.92 (s, 6H, CH_3_), 4.76 (d, *J *= 2.4 Hz, 2H, CH_2_), 7.80 (d, *J *= 1.5 Hz, 2H, ArH), 8.30 (t, *J *= 1.5 Hz, *J *= 1.2 Hz, 1H, ArH).

*(3-Hydroxymethyl-5-prop-2-ynyloxy-phenyl)methanol *(**18**). Compound **17 **(0.500 g, 2.01 mmol) was dissolved in THF (35.0 mL), and degassed. LiAlH_4_ (12.1 mmol) was added to the solution. The reaction mixture was stirred for 6 h, and the reaction was confirmed by TLC (EtOAc–*n*-hexane = 1:1). Then, water was added to the solution in order to quench the reaction. The solution was dried over MgSO_4_, and MgSO_4_ was removed by filtration. The solution was evaporated to yield white solid; Yield 0.425 g, 99%. ^1^H-NMR (300 MHz, r.t., CD_3_OD): δ/ppm 2.91 (t, *J *= 2.7 Hz, *J *= 2.4 Hz, 1H, C≡CH), 4.57 (s, 4H, CH_2_OH), 4.72 (d, *J *= 2.4 Hz, 2H, CH_2_), 6.89 (s, 2H, ArH), 8.30 (d, *J *= 0.6 Hz, 1H, ArH).

*1,3-Bischloromethyl-5-prop-2-ynyloxy-benzene *(**19**). Compound **18** (0.425 g, 2.21 mmol) was dissolved in CH_2_Cl_2_, and SOCl_2 _was added to the solution with catalytic amount of DMF at 0 °C. The reaction mixture was stirred for overnight, and the reaction was confirmed by TLC (EtOAc–*n*-hexane = 1:1). The reaction mixture was poured into ice-water, and extracted with CHCl_3_. The organic phase was washed with water, and dried over MgSO_4_. MgSO_4_ was removed by filtration, and the solution was evaporated to yield white solid; Yield. 0.462 g, 91.2%. ^1^H-NMR (300 MHz, r.t., CDCl_3_): δ/ppm 2.52 (t, *J *= 2.4 Hz, *J *= 2.4 Hz, 1H, C≡CH), 4.53 (s, 4H, CH_2_), 4.70 (d, *J *= 2.4 Hz, 2H, CH_2_), 6.95 (d, *J *= 1.5 Hz, 2H, ArH), 7.02 (t, *J *= 0.6 Hz, *J *= 0.6 Hz, 1H, ArH).

***G1****-**Cl ***(**20**). Compounds **15** (95.0 mg, 0.391 mmol) and 1**9** (0.420 g, 1.83 mmol) was dissolved in DMF (3 mL). To the reaction mixture, CuI (111 mg, 0.586 mmol) was added and suspended. DIEA (100 mL, 0.588 μmol) was added to the solution. The solution was stirred for 24 h. The reaction mixture was evaporated, and the residue was purified by column chromatography (CHCl_3_–MeOH = 60:1) to yield white solid; Yield 0.268 g, 73.7%. ^1^H-NMR (300 MHz, r.t., CDCl_3_): δ/ppm 4.50 (s, 12H, CH_2_), 5.20 (s, 6H, CH_2_), 5.46 (s, 6H, CH_2_), 6.94 (s, 6H, ArH), 6.99 (s, 3H, ArH), 7.10 (s, 3H, ArH), 7.54 (s, 3H, C=CH). 

***G1****-**N_3_*** (**21**). Compound **20 **(0.268 g, 0.288 mmol) was dissolved in DMF (10 mL). NaN_3_ (3.63 mmol) was added to the solution, and the solution was stirred at 50 °C for overnight. The solution was evaporated, and the residue was dissolved in CHCl_3_. The organic phase was washed with water for three times. The solution was dried with MgSO_4_, and MgSO_4_ was removed by filtration. The residue was purified by column chromatography (CHCl_3_–MeOH = 100:1) to yield white solid; Yield 0.260 g, 93.1%. ^1^H-NMR (300 MHz, r.t., CDCl_3_): δ/ppm 4.29 (s, 12H, CH_2_), 5.21 (s, 6H, CH_2_), 5.45 (s, 6H, CH_2_), 6.85 (s, 3H, ArH), 6.88 (s, 6H, ArH), 7.13 (s, 3H, ArH), 7.55 (s, 3H, C=CH). ^1^H-NMR (300 MHz, r.t., DMF-d_7_): δ/ppm 4.50 (s, 12H, CH_2_) 5.23 (s, 6H, CH_2_), 5.71 (s, 6H, CH_2_), 7.05 (s, 3H, ArH), 7.10 (s, 6H, ArH), 7.45 (s, 3H, ArH), 8.39 (s, 3H, C=CH). ^13^C-NMR (75 MHz, r.t., CDCl_3_): δ/ppm 53.4, 54.4, 62.1, 114.3, 120.6, 122.9, 127.6, 136.8, 137.7, 144.4, 158.8. ESI-MS: m/z 969.9.0 [M+H]^+^, 992.0 [M+Na]^+^.

*pNP 6-Sulfo-GlcNAc hexamer(**G1**:**2******2****)*. Compounds **21 **(6.15 mg, 6.34 μmol) and **10 **were dissolved in DMF (400 μL), and an aqueous solution of sodium ascorbate (6.02 mg, 0.0304 mmol) and CuSO_4_ (2.40 mg, 0.0150 mmol) was added to the solution. The reaction mixture was stirred for 3 days. The solvent was evaporated and the residue was purified by reversed phase chromatography (H_2_O–MeOH = 2:1) to yield a white solid; Yield 7.70 mg, 28.2%. ^1^H-NMR (300 MHz, r.t., DMF*-d_7_*): δ/ppm 1.26–1.28 (m, 12H, CH_2_), 1.88 (s, 18H, OCOCH_3_), 1.96 (t, *J *= 7.5Hz, *J *= 7.5 Hz, 12H, CH_2_), 2.41 (t, *J *= 7.5 Hz, *J *= 7.8 Hz, 12H, CH_2_), 3.92 (dd, *J*_H1-H2_ = 9.5Hz, *J*_H2-H3_ = 9.0 Hz, 6H, H2), 4.03–4.06 (m, 6H, H5), 4.07–4.11 (m, 6H, H3), 4.19–4.26 (m, 6H, H4), 5.02 (d, *J*_H1-H2_ = 9.0 Hz, 6H, H1), 5.16 (s, 6H, CH_2_), 5.29 (broad, 6H, H6), 5.36 (broad, 6H, H6), 5.57 (s, 12H, CH_2_), 5.66 (s, 6H, CH_2_), 6.91 (s, 3H, ArH), 6.94 (d, 12H, *J*_o‑m_ = 9.0Hz, Ph-o), 7.03 (s, 6H, ArH), 7.44 (s, 3H, ArH), 7.58 (d, 12H, *J*_o-m_ = 9.0Hz, Ph-m), 7.94 (s, 6H, C=CH), 8.40 (s, 3H, C=CH). ^13^C-NMR (75 MHz, r.t., DMF*-*d_7_): δ/ppm 23.5, 28.2, 30.6, 31.0, 47.9, 58.4, 59.8, 61.8, 67.1, 71.7, 76.8, 80.4, 81.2, 106.0, 115.2, 119.8, 122.5, 126.0, 130.6, 133.6, 140.1, 143.1, 144.2, 159.4, 164.6, 175.5, 176.4. ESI-MS: *m/z* 822.9 [M+H+4Na]^5+^.

*G2-Cl *(**23**). G1-N_3_ (**21**, 0.0597 g, 0.0616 mmol) and **19** (0.114 g, 0.498 mmol) was dissolved in DMF (750 μL). CuI (21.0 mg, 0.110 mmol) was added to the solution and suspended. DIEA was added to the solution, and the solution was stirred for 24 h at room temperature. The reaction was confirmed by TLC (CHCl_3_–MeOH = 10:1), and the residue was purified by column chromatography (CHCl_3_–MeOH = 40:1) to yield a white solid; Yield 43.5 mg, 30.1%. ^1^H-NMR (300 MHz, r.t., CDCl_3_): δ/ppm 4.45 (s, 24H, CH_2_), 4.99 (s, 6H, CH_2_), 5.07 (s, 12H, CH_2_), 5.12 (s, 6H, CH_2_), 5.37 (s, 12H, CH_2_), 6.73 (s, 3H, ArH), 6.75 (s, 6H, ArH), 6.88 (s, 12H, ArH), 6.94 (s, 6H, ArH), 7.05 (s, 3H, ArH), 7.55 (s, 3H, C=CH), 7.62 (s, 6H, C=CH).

*G2-N_3 _*(**24**)*. ***23** (0.0435 g, 0.0186 mmol) was dissolved in DMF (10 mL). NaN_3_ (0.0201 g, 0.334 mmol) was added to the solution, and stirred at 50 °C for overnight. The solution was evaporated and the residue was dissolved in CHCl_3_. The organic phase was washed with water three times. The organic phase was dried over MgSO_4_, and MgSO_4_ was filtrated. The residue was purified by column chromatography (CHCl_3_–MeOH = 40:1) to yield a white solid; Yield 0.253 g, 56.3%. ^1^H-NMR (300 MHz, r.t., CDCl_3_): δ/ppm 4.27 (s, 24H, CH_2_), 4.5.04 (s, 6H, CH_2_), 5.16 (s, 12H, CH_2_), 5.17 (s, 6H, CH_2_), 5.40 (s, 12H, CH_2_), 6.76 (s, 3H, ArH), 6.78 (s, 6H, ArH), 6.83 (s, 6H, ArH), 6.86 (s, 12H, ArH), 7.08 (s, 3H, ArH), 7.54 (s, 3H, C=CH), 7.61 (s, 6H, C=CH). ^ 1^H-NMR (300 MHz, r.t., DMF-*d_7_*): δ/ppm 4.48 (s, 24H, CH_2_), 5.15 (s, 6H, CH_2_), 5.24 (s, 12H, CH_2_), 5.66 (s, 6H, CH_2_), 5.69 (s, 12H, CH_2_), 7.03 (s, 6H, ArH), 7.06 (s, 9H, ArH), 7.09 (s, 12H, ArH), 7.44 (s, 3H, ArH), 8.35 (s, 3H, C=CH), 8.41 (s, 6H, C=CH). ^13^C-NMR (75 MHz, r.t., CDCl_3_): δ/ppm 53.3, 53.6, 54.3, 61.7, 62.0, 114.2, 114.6, 120.0, 120.6, 123.2, 123.5, 127.7, 136.7, 137.2, 137.7, 143.6, 144.1, 158.7, 159.0. MALDI-TOF-MS: *m/z* 2447.2 [M+Na]^+^.

*pNP 6-Sulfo-GlcNAc dodecamer *(**G2**:**25**). Compounds **24 **(8.00 mg, 3.30 μmol) and **10 **were dissolved in DMF (350 μL). The aqueous solution (83 μL) of sodium ascorbate (6.28 mg, 31.7 mmol) and CuSO_4_ (2.52 mg, 0.0158 mmol) was added to the solution. The reaction mixture was stirred for 3 days. The reaction was confirmed by TLC (H_2_O–MeOH = 3:1). The solution was evaporated and the residue was purified by reversed phase column chromatography (H_2_O–MeOH = 3:1) to yield a white solid; Yield 6.30 mg, 21.1%. ^1^H-NMR (500 MHz, r.t., DMF-*d_7_*): δ/ppm 1.26–1.28 (m, 24H, CH_2_), 1.88 (s, 36H, OCOCH_3_), 1.94–199 (m, 24H, CH_2_), 2.40 (t, *J *= 7.5 Hz, *J *= 7.0 Hz, 24H, CH_2_), 3.78 (broad, 12H, H5), 3.87–3.90 (m, 12H, H2), 4.06–4.10 (m, 12H, H3), 4.23–4.26 m, 12H, H4), 4.25 (m, 6H, H4), 5.01 (d, *J*_H1-H2_ = 8.5 Hz, 12H, H1), 5.16–5.18 (m, 12H, H6), 5.22–5.24 (broad, 6H, CH_2_), 5.31–5.34 (m, 6H, H6), 5.36 (broad, 6H, H6′), 5.53 (s, 24H, CH_2_), 5.56 (s, 12H, CH_2_), 5.58 (s, 12H, CH_2_), 5.61–5.66 (broad, 6H, CH_2_), 6.88 (s, 6H, ArH), 6.96–6.98 (overlap, 24H, Ph-o), 6.96–6.98 (overlap, 9H, ArH), 7.03(s, 12H, ArH), 7.44 (broad, 3H, ArH), 7.58 (d, 2H, *J*_o-m_ = 8.1 Hz, Ph-m), 7.94 (s, 12H, C=CH), 8.35 (s, 3H, C=CH), 8.35 (s, 6H, C=CH). ^13^C-NMR (75 MHz, r.t., DMF-*d_7_*): δ/ppm 22.6, 28.2, 30.6, 31.1, 58.4, 61.3, 61.8, 67.1, 71.7, 76.8, 80.3, 81.2, 82.3, 91.5, 106.0, 115.4, 120.0, 122.5, 126.0, 127.8, 130.0, 140.1, 143.2, 143.4, 144.2, 148.7, 155.4, 156.6, 156.6, 164.6, 164.7, 176.4, 181.0. ESI-MS *m/z* 1238.0 [M+H+6Na]^7+^.

### 3.4. Lectin Recognition Assay

Each sugar dendrimer was dissolved in PBS (pH 7.4) at a concentration of 8.00 μM. The lectin (FITC-WGA) was also dissolved in PBS (pH 7.4) at a concentration of 0.100 μM. To a solution of WGA (300 μL) were sequentially added aliquots of the sugar dendrimer solutions (3, 3, 5, 5, 5, 5, 10, 10, 10, 10, 10, 20, 20 and 20 μL) at 25 °C. Fluorescent measurements were taken 5 min after the addition of each aliquot. Fluorescence spectra of the FITC were measured with an excitation wavelength at 490 nm and an emission wavelength of 510 nm. The PBS buffer without a sugar dendrimer was also added in the same way as a reference. Changes in the fluorescence of the sugar-protein interaction were calculated by subtracting two of fluorescence intensities (*i.e.*, fluorescence change by sugar protein interaction = fluorescence change by addition of the sugar dendrimer solution–fluorescence change by the addition of the reference PBS solution). Changes in the fluorescence were plotted according to the following formula [19,20]:


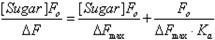


where [Sugar], K_a_, F_o_, ΔF, and ΔF _max_ represent the sugar concentration of the dendrimer solution (M), the association constant (M^−1^), the initial fluorescence intensity, the fluorescence change, and the maximum fluorescence change, respectively.

### 3.5. *In Vitro* Amyloid Formation of Aβ (1-42) and ThT Fluorescence Assay [14]

Aβ (1-42) was dissolved in a 0.02% ammonia solution at a concentration of 200 μM. Any aggregates formed were removed by centrifugation using a CS120FX centrifuge (Hitachi, Tokyo, Japan) for 3 h at 16,000 g and 4 °C. The supernatant was mixed with phosphate buffer (20 mM phosphate buffer, pH 7.4, 100 mM NaCl) to a final peptide concentration of 20 μM. The peptide solution was incubated with each sugar additive at 37 °C. The concentration of the sugar additives was 200 μM. Amyloid fibril formation was evaluated by the fluorescence emission of ThT. Following a period of incubation at 37 °C, a 5 μL solution of Aβ in buffer was added to 300 μL of the ThT solution (50 mM in the same phosphate buffer). Following a period of 10 s, the fluorescence intensity was measured at an excitation wavelength of 450 nm and an emission wavelength of 482 nm.

### 3.6. AFM Measurements

The Aβ peptide (1-42) was incubated in phosphate buffer (20 mM phosphate buffer, 100 mM NaCl) at 37 °C for 12 h, with the peptide and the sugar additives being added at a concentration of 20 μM. After the incubation of the sample, a 5 μL portion of the sample solution was placed on freshly cleaved mica and dried under N2. The mica substrates were then washed with 100 μL of MilliQ water.

## 4. Conclusions

A series of novel glycodendrimers containing sulfonated GlcNAc have been successfully synthesized using click chemistry. The glycodendrimers of **G1** and **G2** formed large glycoside clusters and exhibited strong affinities to proteins of lectin and Aβ (1-42) as a consequence of the multivalent effect. **G1 **showed the highest level of affinity to proteins because of the self-assembling properties of the dendrimer. **G1** and **G2** both strongly inhibited Aβ aggregation and β-sheet conformation. 

Click chemistry was used in the current paper to successfully synthesize the glycodendrimers. The procedure was facile and proceeded without the need for protecting group chemistry. Work focused on the fabrication of functional cluster of glycosides with saccharides suitable for application in pathogens is currently underway in our laboratories. 
